# hElp3 Directly Modulates the Expression of HSP70 Gene in HeLa Cells via HAT Activity

**DOI:** 10.1371/journal.pone.0029303

**Published:** 2011-12-21

**Authors:** Fen Li, Jixian Ma, Yu Ma, Yanyan Hu, Shujuan Tian, Richard E. White, Guichun Han

**Affiliations:** 1 College of Life Science, Henan Normal University, Xinxiang, China; 2 Department of Veterinary Physiology and Pharmacology, Texas A&M University, College Station, Texas, United States of America; 3 Department of Pharmacology and Toxicology, Georgia Health Sciences University, Augusta, Georgia, United States of America; Tulane University Health Sciences Center, United States of America

## Abstract

Human Elongator complex, which plays a key role in transcript elongation *in vitro* assay, is incredibly similar in either components or function to its yeast counterpart. However, there are only a few studies focusing on its target gene characterization *in vivo*. We studied the effect of down-regulation of the human elongation protein 3 (h*ELP3*) on the expression of *HSP70* through antisense strategy. Transfecting antisense plasmid p1107 into HeLa cells highly suppressed h*ELP3* expression, and substantially reduced expression of *HSP70* mRNA and protein. Furthermore, chromatin immunoprecipitation assay (ChIP Assay) revealed that hElp3 participates in the transcription elongation of *HSPA1A* in HeLa cells. Finally, complementation and ChIP Assay in yeast showed that hElp3 can not only complement the growth and slow activation of *HSP70* (*SSA3*) gene transcription, but also directly regulates the transcription of *SSA3*. On the contrary, these functions are lost when the HAT domain is deleted from hElp3. These data suggest that hElp3 can regulate the transcription of *HSP70* gene, and the HAT domain of hElp3 is essential for this function. These findings now provide novel insights and evidence of the functions of h*ELP3* in human cells.

## Introduction

Elongator was first identified as a component of an RNAPII holoenzyme isolated from yeast chromatin [1]. Its catalytic subunit, Elp3, has domains characteristic of the GCN5 family of histone acetyltransferases (HATs) [2]. The GCN5 family of HATs is part of a larger superfamily of enzymes capable of acetyl transfer to amine-containing substrates (referred to as GNATs, for GCN5-like N-acetyltransferases). Deletion of Elp3 results in decreased levels of multiple acetylated histone H3 and H4 in chromatin in vivo [3], and the steady-state level of H3 and H4 acetylation in the coding region of the gene has a positive correlation with its transcription activity [4]. Combining an *elp3Δ* mutation with histone H3 and H4 tail mutations confers lethality or sickness, supporting a role for Elongator in chromatin remodeling in vivo [5]. Elongator is associated with the nascent RNA emanating from elongating RNAPII along the coding region of several yeast genes [6]. The central region of Elp3 shares significant sequence homology to the Radical SAM superfamily (Members of this superfamily are related by the cysteine motif CxxxCxxC, which nucleates the [4Fe–4S] cluster found in each) [7]. Subsequent experiments have demonstrated that it indeed contains the Fe_4_S_4_ cluster, which characterizes the Radical SAM superfamily and binds SAM, suggesting that Elp3, in addition to its HAT activity, has a second as yet uncharacterized catalytic function [8].

Purified human Elongator can exist in two forms: a six-subunit complex, holo-Elongator, which has histone acetyltransferase activity directed against histone H3 and H4, and a three-subunit core form, which does not have histone acetyltransferase activity despite containing the catalytic Elp3 subunit. The holo-enzyme can associate with RNAPII both in solution and in elongation complexes [9], and is believed to serve a role in RNAP II-associated chromatin remodeling during transcript elongation. Immunodepletion of the Elongator subunits from HeLa nuclear extracts reduced the ability of these extracts to transcribe chromatin templates in vitro, providing biochemical support for this hypothesis [10]. We have recently demonstrated that hElp3 can significantly complement the defects of *elp3Δ* strain, and that HAT activity is essential for this function in vivo. The results of specific lysine mutations in histone H3 and H4 assay in yeast imply a link between the acetylation of specific sites in nucleosomal histones and the regulation of transcription elongation by human Elp3 [11]. In human cells Elongator complex can be detected at several genes by chromatin immunoprecipitation (ChIP) [12]–[14]. Metivier et al. [12] used ChIP assay to provide evidence for the idea that Elongator only arrives at a gene after RNAPII hyperphosphorylation has taken place. Others found that Elonagtor is primarily associated with the coding regions of genes [13], [14]. Recent experiments have shown that Elongator is associated with the coding region of genes, and affects histone acetylation and transcript elongation in human cells, transcription defects may underlie familial dysautonomia [14]. However, the candidate target gene and the mechanisms by which human Elongator regulates its expression in human cells still remain poorly understood.

Human and yeast Elongator share many functions. We have shown that yeast *HSP70 (SSA3)* is the target gene of yElp3, and that hElp3 can complement the defects in growth and activation of induced genes of *elp3Δ* strain [9], [15]. We hypothesize that human *HSP70* is one of the target genes of hElp3. Now, we report the effect of antisense down regulation of hElp3 on *HSP70* expression. We demonstrate that plasmid p1107, containing reverse inserted HAT-deletion Elp3, can significantly depress the transcription of hElp3. Northern blot and Western blotting analyses reveal that down regulation of hElp3 expression results in dramatically suppressed expression of *HSP70* mRNA and protein in HeLa cells. ChIP assay indicates that hElp3 participates in the transcript elongation of *HSPA1A.* Complement and Chip assay in yeast showed that hElp3 can also regulate the *HSP70* (*SSA3*) transcription but the HAT domain deleted mutant hElp3HAT^−^ cannot. Our results provide evidence for *HSP70* being the target gene of hElp3 and the HAT domain of hElp3 plays crucial role in transcription regulation.

## Results

### Reduction of hELP3 mRNA level in HeLa cells

**Figure 1:**
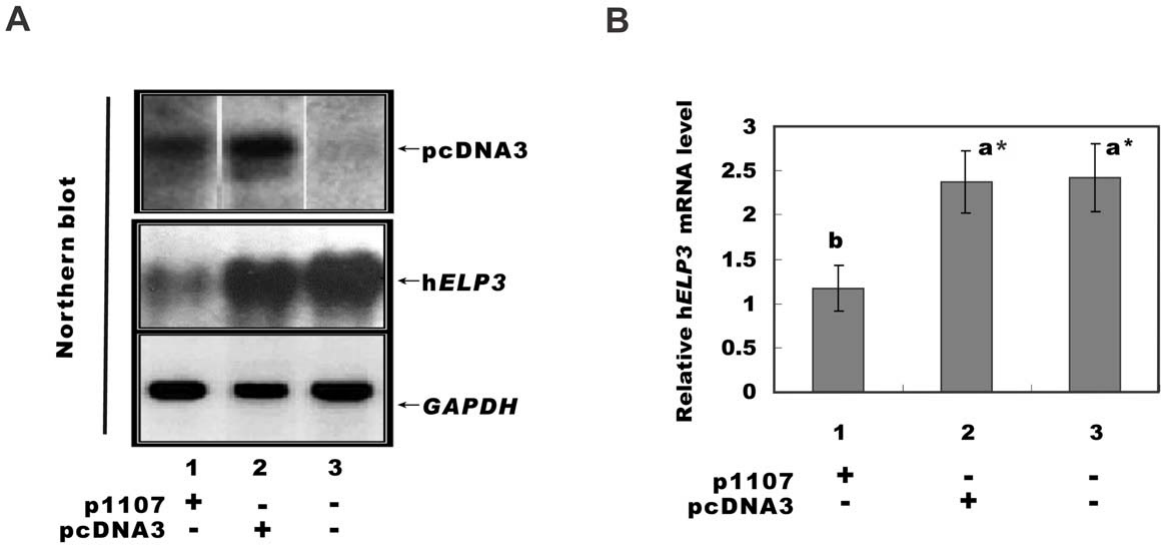
Expression of *ELP3* gene in HeLa cells. A: Northern blot products of exogenous gene. The arrows indicate the bands of Northern blot products of exogenous gene (upper arrow), *ELP3* (middle arrow) and the internal control of *GAPDH* (lower arrow). B: Relative expression level of Elp3 normalized to *GAPDH* via photodensitometric analysis of Northern blot products. A significant difference is indicated by * (p<0.01). Non-transfected HeLa cells were used as control.

Northern blot studies verified the validity of p1107 function in HeLa cells. As shown in Fig. 1A, exogenous gene expression was detected in transfection groups (p1107 and pcDNA3), but not in non-transfected HeLa cells. This suggests that the exogenous gene was transfected into HeLa cell and expressed successfully. A semi-quantitative procedure was used to analyze changes in transcription of Elp3. The Northern bolt products in Fig. 1A were quantified by photodensitometry, and expressed as a ratio between the readings of h*ELP3* and that of *GAPDH* in Fig. 1B. As can be seen, transfection of p1107 into HeLa cells dramatically reduced endogenous expression of *ELP3* (P<0.01). In contrast, pcDNA3 had no effect, as the expression of *ELP3* was nearly the same as that of the non-transfected HeLa cells (P>0.05) (Fig. 1B). This indicates that p1107 antisense fragment is effective in HeLa cells.

### Antisense Down regulation of the Elp3 reduced the *HSP70* mRNA level in HeLa cells

**Figure 2:**
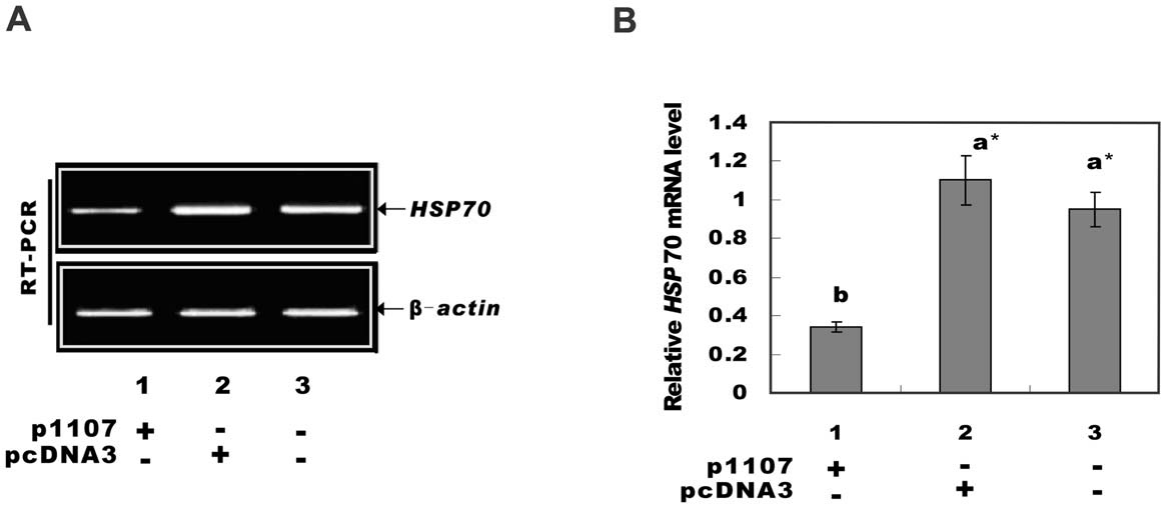
Expression of *HSP70* in HeLa cells. A. Cells were heat shocked at 42°C for 1 h. Total RNA was extracted after a 2 h recovery at 37°C. Arrows indicate bands of RT-PCR products of *HSP70* (upper arrow) and the internal control of *β-actin* (lower arrow). B. Relative expression level of *HSP70* normalized to *β-actin* ratio via photodensitometric analysis of RT-PCR products. *significant (p<0.01).

Semi-quantitative RT-PCR procedure probed the effect of hElp3 down regulation on *HSP70* transcription in HeLa cells. The RT-PCR products were resolved by agarose gel electrophoresis (Fig. 2A), quantified by photodensitometry, and expressed as a ratio between the readings of *HSP70* and that of *β-actin* (Fig. 2B). Compared with non-transfected HeLa cells, *HSP70* mRNA level was significantly reduced in cells transfected with p1107 (P<0.01), whereas transfecting pcDNA3 had no effect (P>0.05). These findings indicate that down regulation of *ELP3* results in the dramatically reduced transcription of *HSP70* in HeLa cells. This result implicated the involvement of hELP3 in *HSP70* gene regulation in human cells.

### Antisense down-regulation of the Elp3 reduced the Hsp70 protein level in HeLa cells

**Figure 3:**
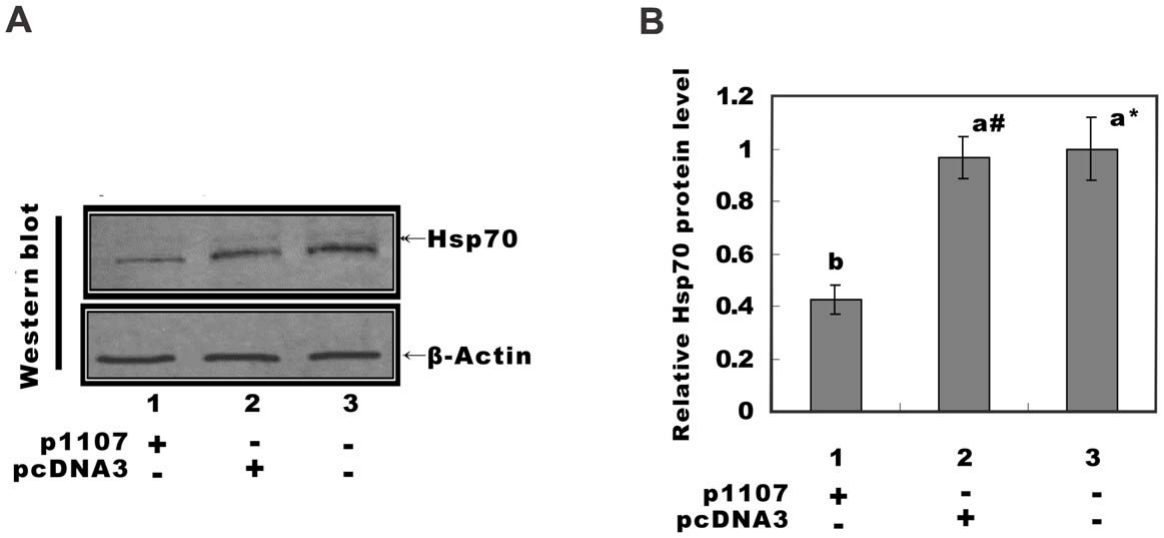
Hsp70 protein level in HeLa cells. A: Western blot results of Hsp70 protein level in HeLa cells. HeLa cells were heat shocked for 1 Hour at 42°C and recovered at 37°C for 8–16 hours. Total cell extracts (1×10^6^) were subjected to Western-blot analyses for Hsp70 level. B: Quantitative densitometric analyses of band intensities. A significant difference is indicated by either *(p<0.01) or # (p<0.05). Non-transfected HeLa cells were used as control.

Western blot was performed to detect the effect of down regulation of *ELP3* on the Hsp70 protein level. (Fig. 3A). As seen in Fig. 3B, the content of Hsp70 in the p1107 transfection group was much lower than that of pcDNA3 and non-transfection control groups (p<0.01 and P<0.05 respectively), but there was no significant difference between pcDNA3 transfection group and non-transfected control group (P>0.05). These results indicate that effectively down regulating the expression of *ELP3* can also dramatically reduce the Hsp70 protein level in HeLa cells.

### hELP3 directly regulates the transcript elongation of *HSPA-1A* gene in HeLa cells

ChIP assay was used to determine transcription factor interaction with candidate target gene, and monitor the presence of histones with post-translational modifications at specific genomic locations [16], [17]. Briefly, in ChIP protein-DNA complexes are crosslinked, immunoprecipitated, purified, and amplified for gene- and promoter-specific analysis of known targets using PCR or real time PCR or sequencing or for the identification of new target sequences.

**Figure 4:**
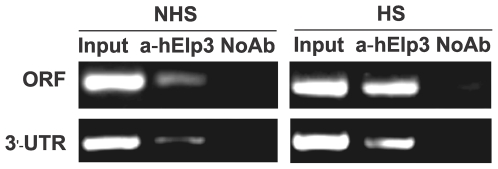
Elp3 present along the *HSPA1A* gene in HeLa cells. HeLa cells were heat-shocked for 2 h at 42°C (HS), or left at 37°C (NHS), and chromatin immunoprecipitation assays with anti-hElp3 antibody were carried out. Immunoprecipitated DNA was analyzed by polymerase chain reaction using primers specific to the open reading frame (ORF) and 3′ untranslated region (UTR) of the *HSPA-1A* gene. Products were analyzed by agarose gel electrophoresis followed by ethidium bromide staining. Input: total input DNA or sonicated genome DNA as template, The “input” sample is an aliquot of chromatin that has not been immunoprecipitated but has been crosslinked, sonicated, and processed in a manner identical to the experimental samples.; a-hElp3: hElp3 antibody immunoprecipitated DNA as template; NoAb: primary antibody was omitted.

To investigate whether hElp3 directly regulates the *HSP70* gene expression, ChIP assay was performed in conjunction with PCR. A large part of the data published on human Hsp70 family deals with the major stress-inducible members of the family, Hsp70-1a and 1b (Homo sapiens heat shock 70 kDa protein 1a and 1b, Encoded by *HSPA1A* and *HSPA1B* respectively, collectively called Hsp70-1). The basal expression of *HSPA1A* and *HSPA1B* mRNAs differ slightly in most tissues, with somewhat higher expression of *HSPA1A* in most tissues and cell types [18]. This study focused on *HSPA1A*. Results demonstrated that hElp3 was present at both the ORF (open reading-frame) and 3′UTR (3′ untranslated region) of *HSPA1A* in HS (heat shock) and NHS (non heat shock) HeLa cells (Fig. 4), indicating that hElp3 can bind with the *HSAPA1A* templates during the whole transcription elongation. These findings suggest that hElp3 directly participates in regulation of the basal and inducible transcript elongation of *HSPA1A*.

### HAT activity of hElp3 is essential for regulating the transcript elongation of *HSP70* gene

**Figure 5:**
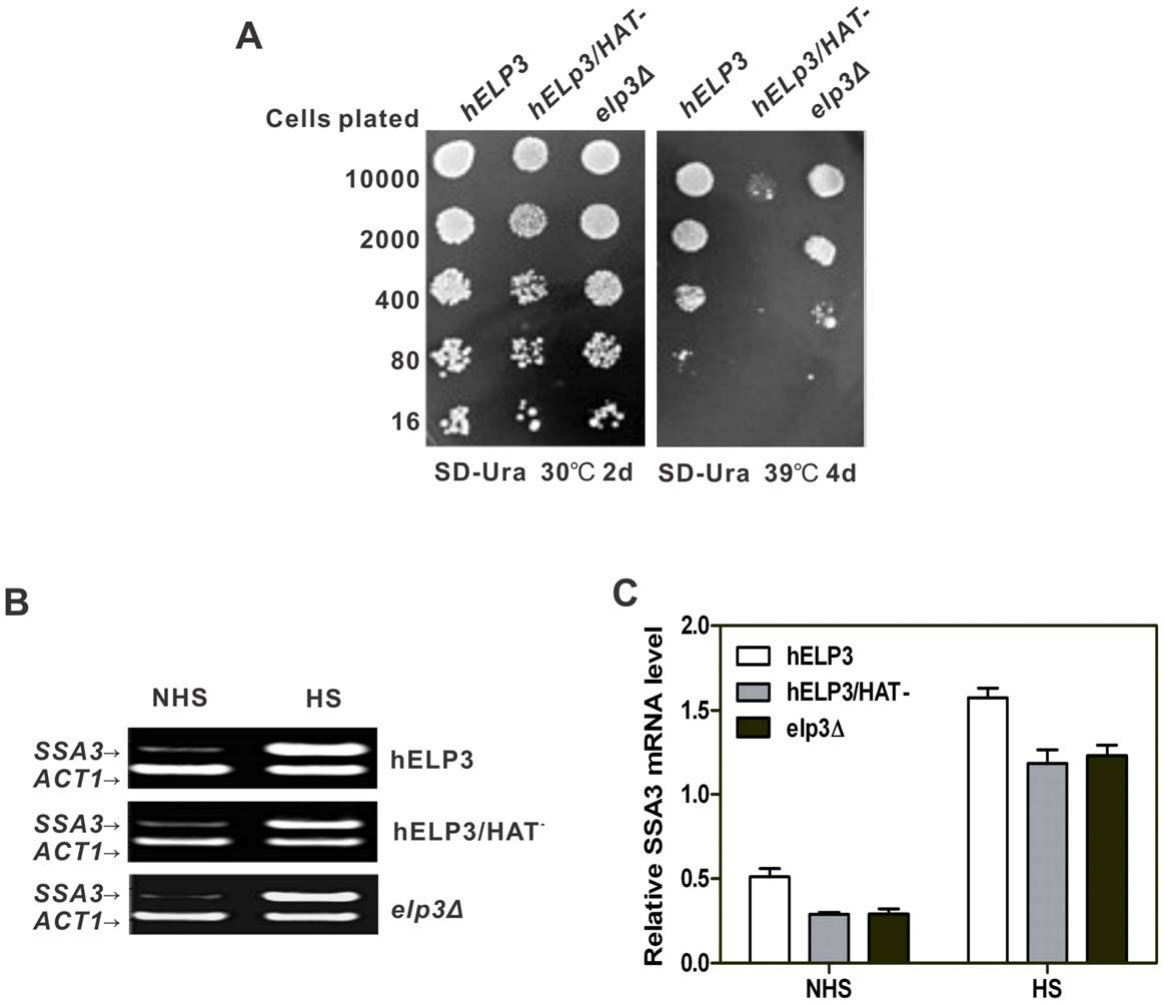
Human Elp3 can complement the growth defect and slow activation of *SSA3* in *elp3Δ* cells and the HAT domain is essential for this function. A: Complementtation tests of *hELP3*, *hELP3/HAT^−^* in the *elp3Δ* strain. The plasmids *hELP3*, *hELP3/HAT^−^* were transformed into the *elp3Δ* strain, and the transformants were plated in a dilution series onto SD-Ura medium and grown at 30°C for 2 days, the transformants were checked for temperature sensitivity after incubation at 39°C for 4 days. B: Complementation of the slow activation of *SSA3* in *elp3Δ* cells. Cells were grown at 26°C before transfer to 37°C to activate the *SSA3* gene. Total RNA was extracted for RT-PCR at 0 or 10 min after heat shocked. The RT-PCR products derived from transcripts of *SSA3* and *ACT1* (the internal control) are shown in the left panel. The panels on the right show the results of the quantitative densitometric analyses of the band intensities at each time-point.

We have previously reported that *hELP3* can complement the defects of slow-growth and slow-activation of yeast *HSP70* transcription of *elp3Δ* strain [11]. Here we show that hElp3HAT^−^, a deletion mutant of lacking catalytic motif B (residues 527–547) of the HAT domain of hElp3, did not complement the slow-growth defects of the *elp3Δ* strain (Fig. 5A). This finding indicates that the highly conserved HAT function of the hElp3 subunit of the Elongator complex is essential for complementation of the slow-growth phenotype.

In order to know whether HAT activity is required for the function of transcriptional activation, we therefore tested the complementation capacity of hElp3 and HAT domain deleted hElp3-expressing plasmids with respect to the activation of *SSA3* in *elp3Δ* strain. A semi-quantitative RT-PCR procedure was used to analyze changes in the transcription of *SSA3* genes upon transfer to inducing conditions. The RT-PCR products were resolved by agarose gel electrophoresis (Fig. 5B), quantified by densitometry, and the amount of *SSA3* transcript was expressed relative to that of the *ACT1* (a housekeeping gene used as internal reference) mRNA (Fig. 5C). As shown in Fig. 5C. Transformation of the *hELP3* gene into *elp3Δ* cells reduced the delay activation of *SSA3* (*hELP3*/*elp3Δ*; P<0.05). But the *hELP3HAT^−^* transformants displayed no complementation function, as the level of *SSA3* expression was nearly the same as that in the *elp3Δ* strain (*hELP3HAT^−^*/*elp3Δ*; P>0.05). Therefore, the human Elp3 HAT activity is critical for both growth and transcriptional activation of *SSA3*.

**Figure 6:**
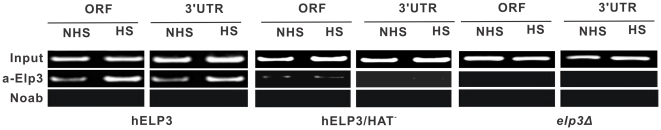
Human Elp3 participates in regulation the transcription of *SSA3* in yeast and its HAT domain is essential for this function. The plasmids containing *hELP3*, *hELP3/HAT* ^−^ were transformed into the *elp3Δ* strain, transformant cells were heat-shocked for 10 min at 37°C (HS), or left at 30°C (NHS), and chromatin immunoprecipitation assays with anti-hElp3 antibody were carried out. Immunoprecipitated DNA was analyzed by polymerase chain reaction using primers specific to the open reading frame (ORF) and 3′ untranslated region (UTR) of the *SSA3* gene. Products were analyzed by agarose gel electrophoresis followed by ethidium bromide staining. Input: total input DNA or sonicated genome DNA as template, The “input” sample is an aliquot of chromatin that has not been immunoprecipitated but has been crosslinked, sonicated, and processed in a manner identical to the experimental samples; a-hElp3: hElp3 antibody immunoprecipitated DNA as template; NoAb: primary antibody was omitted.

To investigate whether HAT activity of hElp3 is also essential for direct regulating the *SSA3* gene expression, ChIP assay was performed in conjunction with PCR. As seen in left two lanes of Fig. 6, hElp3 was present at both the ORF and 3′UTR of *SSA3* in HS and NHS hElp3 transformant cells, indicating that hElp3 can bind with the *SSA3* templates during the whole transcription elongation. But, hElp3HAT^−^ is almost undetectable at both ORF and 3′UTR of *SSA3* in HS and NHS *hElp3HAT^−^* transformed *elp3Δ* cells. These findings suggest that hElp3 directly participates in regulation of the basal and inducible transcript elongation of *SSA3* in yeast and the HAT activity of hElp3 is essential for this function.

## Discussion

### hELP3 directly participates in regulation of *HSP70* gene transcript elongation in human cells, and the HAT activity is crucial for this function

We have previously reported that *hELP3* is a histone H3/H4 acetyltransferase and can activate yeast *HSP70* transcription by complementation [11]. Our recent ChIP assay [15] demonstrated that yElp3 directly participates in yeast *HSP70* (*SSA3*) transcript elongation. Therefore, we hypothesize that hElp3 is involved in *HSP70* transcription in human cells. In the present study we have employed antisense strategy to demonstrate that down regulation of *hELP3* can result in a dramatic reduction of *HSP70* mRNA and protein (Fig. 2 and Fig. 3). These data provide evidence in support of the previously proposed model for Elongator function, which has been described to assist RNAPII traveling through the *HSP70* chromatin during transcript elongation[14] (Close et al. 2006). ChIP assay provided evidence for a role of hElp3 in directly regulating the basal and inducible transcription of *HSPA1A*. These data support the previous reports that Elongator is associated with the coding regions of genes [13], [14]. Defects in the *hELP1* subunit of human Elongator affect transcriptional elongation of the genes encoding proteins implicated in cell motility [14], thus indicating that human Elongator may be involved in transcriptional regulation of a range of different genes. Results from this study strongly point to a function for the human Elongator in transcription of *HSP70* in HeLa cells. Since hElp3 can complement the defects of slow-growth and slow-activation of *elp3 Δ* cells, we wanted to know whether the conserved HAT domain is involved in this function. Our findings (Figs. 5 and 6) demonstrated that even though hElp3 can alleviate the slow-growth and slow *SSA3* activation defects of yeast *elp3Δ* cells at high temperature and participate in the transcript elongation of *HSP70* (*SSA3*), the HAT deleted mutant hElp3 has almost no complement function at all. ChIP assay further demonstrated that the HAT-deleted mutant hElp3 cannot be found at ORF of 3′-UTR in HS (heat shock) or NHS (non heat shock) yeast cells. These findings indicate that the conserved HAT domain of hElp3 plays a key role in regulation of *HSP70* transcription, thus dramatically influencing the complementation function of HAT domain deleted mutant. Altogether our findings strongly point to a function of the human Elongator in transcription of *HSP70*, and indicate that HAT activity of Elongator is crucial for this function.

### Complex function of Elongator in human diseases

Elongator has been proposed to participate in three distinct cellular processes in yeast: transcriptional elongation [1], polarized exocytosis and formation of modified wobble uridines in transfer RNA (tRNA) [19], [20]. In Arabidopsis the homolog of the Elp3, Elongata3, has been found to bind to the αβ-tubulin heterodimer, suggesting that **α**-tubulin may be a cytoplasmic target of Elongator acetylase activity [21]. Familial dysautonomia (FD), a disease of the autonomic and sensory nervous systems, involves mutations in the protein IκB kinase complex-associated protein (*IKAP*), which is a component of the human Elongator complex [22]. Without Elp1, Elp3 protein is highly unstable and barely detectable in yeast [23], and Elp3 levels are also significantly reduced in fibroblasts from FD patients [14]. Accordingly, the amount of Elongator complex detected at genes is reduced in FD cells, and cells with lower levels of Elongator also display pronounced cell migration and cell motility defects [14]. Elongator has been found to control the migration and differentiation of cortical neurons through acetylation of alpha-tubulin [24], and variants of the elongator protein 3 (*ELP3*) gene are associated with motor neuron degeneration in brain tissue of human [25]. Human *HSP70* is associated with many human disorders, such as Crohn's diseases [26], diabetes [27], schizophrenia [28], cancer [29], and Parkinson's disease [30]. Elongator complex might be involved in these disease states, but further study of the functional roles of Elongator is warranted.

### The various roles of Elongator in a variety of tissues/species

Recent evidence has linked Elongator to the RNA interference in Drosophila [31], DNA damage response and gene silencing in yeast [32], and global DNA demethylation in mouse zygotes [33]. Several proteins, including Kti12 [34], IKB [35], [36], STAT3 [36], and PCNA [32], have been reported to interact with components of the Elongator complex. While some interactions have been confirmed in different species, most studies have been done in a single species. It is necessary and important to explore the possibility that there might be cell/tissue-specific and species-specific functions of ELP3 impacting expression patterns in various cell types of different species. In this study we demonstrated that hElp3 directly regulates the expression of *HSPA1A* in HeLa cells. Together with a recent study on *HSP-2* in 293T human embryonic kidney epithelial cells [37], our findings strongly point to a function of the human Elongator in transcription of *HSP70* in human cells. We are still far from having a comprehensive view of all Elongator functions, and further evidence from variety species or human cells is needed in order to completely identify and characterize the functions of hElp3. Such studies will be beneficial to helping us better understand the role of Elongator in human diseases.

## Materials and Methods

### Cell culture

The HeLa (CCL-2, ATCC, USA) cells were maintained in DMEM (ATCC, USA) with 10% fetal calf serum (GIBCO USA), 4 mM L-glutamine, 100 µg/ml penicillin and 100 µg/ml streptomycin. Cell cultures were kept at 37°C and under 5% CO_2_ in a humidified culture incubator. For the heat shock experiments, cells were treated at 42°C for 2 h.

### Yeast culture and growth condition

Saccharomyces cerevisiae strain JSY130: *MATa ade2-1 ura3-1 his3-11, 15, leu2-3, 112 trp1-1 can1-100 elp3Δ :: LEU2* was propagated using standard procedures in either rich medium (YPD) and transformed with pRS316, pRS316hELP3 and pRS316hELP3HAT^−^plasmids. Transforants were grown under similar conditions in SD medium without uracil (SD-Ura). Heat-shock induction was performed by growing transformants at 26°C to mid-log phase and then transferred to 37°C water bath and shaking vigourously; once the temperature reached 37°C, the incubation was allowed to continue for an additional 10 min.

### Plasmid constructs

The selected ah1107 fragment (which represses expression of transformed *hELP3* dramatically in *elp3Δ* strain) was amplified as a 5′-*EcoR* I-*Xho* I fragment from pYES2hELP3 using primers listed in Table 1, and cloned into *Sma* I linerized pUC19 to create plasmids pUC19-ah1107. The ah1107 fragment was sub-cloned into *EcoR* I/*Xho* I digestion pcDNA3 (Invitrogen CA) to construct the antisense expression vector p1107. pRS316, pRS316hELP3 and pRS316hELP3HAT^−^ are kept by our laboratory.

**Table 1:** Probe and primer sequence used in this paper.

T7 promotor probe	5′-CAGAGGGATATCACTCAGCATAAT- 3′
*GAPDH* Probe	5′-CAA GCT TCC CGT TCT CAG CC-3′
*hELP3* probe	5′ CGTTTGGGGTGTCTACAGGTGTGT-3′
*HSPA1A* RT	5′-CAAGATCAGCGAGGCCGA-3′/5′-CCCTTGGGACCTGAGC-3′
*β-actin* RT	5′-TTGCGTTACACCCTTTCT-3′/5′-ATGCTATCACCTCCCCCTG-3′
*HSPA1A* 3′-UTR	5′-CTGTTTTTGTTTTGGAGC-3′/5′-ATGGCCTGAGTTAAGTGT-3′
*HSPA1A* ORF	5′-GCCGACAAGAAGAAGGTG-3′/5′-CCCTGAGCCCCGAAGCCG-3′
*SSA3* RT	5′-ATGAAAGGGAGGCAGAA-3′/5′-CCGTAGCACCCGAGTTG-3′
*ACT1* RT	5′-AGCCGTTTTGTCCTTGTT-3′/5′-GCGGTGATTTCCTTTTGC-3′
*SSA3* ORF	5′- CGTATTATCAATGAACCCACTG -3/′5′-GTCTCCTGCGGTAGCCTTA-3′
*SSA3* 3′-UTR	5′-TGCTACGGGAGGTGGAG-3′/5′-CGTGCTGCGGAAACAA-3′

### HeLa cell transfection

A total of 2×10^5^ HeLa cells were seeded into each well of a six-well plate. When the cells reached 70–80% confluence one day later, they were transfected with the appropriate plasmids using lipofectamine (Qiagen, Germany) according to the manufacturer's protocol. After 48 h, stably transfected cells were screened with 380 µg/ml of G418 (Sigma) for two to six weeks. Untransfected HeLa cells and cell clones that were stably transfected with pcDNA3 were established as controls. All transfected cell clones were maintained in the presence of G418 (380 µg/ml) throughout the experiments. These cells were used to assess the effects of human Elp3 on *HSP70* expression in HeLa cells as described below.

### Northern Blot analysis

Total RNAs were extracted from HeLa cells with Trizol reagent (MRC, USA), and the concentration was quantified at OD260 absorbance. For Northern analysis 30 µg of total RNA were heat-denatured at 68°C for 10 min, placed on ice for 5 min, and then applied to a 1% agarose gel in 2.2 M formamide-MOPS buffer. After electrophoresis, the RNA was transferred to a Hybond-N membrane (Roche, Germany), and fixed by baking for 2 h at 70°C. Probes for pcDNA3 and h*ELP3* (shown in table 1) were labeled with [a-^32^P] dCTP (Roche, Germany) by random primer labeling method. Blots were pre-hybridized for at least 3 h, then hybridized for 20 h at 37°C in a buffer containing 50% formamide, 6×SSC, 5×Denhardt's solution [0.1% Ficoll 400, 0.1% BSA, 0.1% polyvinyl- pyrolidone], 5 mM EDTA, 0.5% SDS, and 100 mg/ml sheared denatured herring sperm DNA (Roche, Germany). The filters were washed once at 55°C for 20 min in a solution containing 2×SSC, 1 mM EDTA, and 0.1% SDS, then twice at 50°C for 20 min in a solution containing 0.4×SSC, 1 mM EDTA, and 0.1% SDS. Autoradiographs were exposed at −70°C for 7 days according to the manufacturer's instructions of Kodak films. *GAPDH* was used as control.

### Semi-quantitative RT-PCR for *HSPA1A* mRNA estimation

HeLa cells were heat shocked for 1 h at 42°C and recovered at 37°C for two hours to induce *HSP70*, and total RNAs were extracted from HeLa cells with Trizol reagent. RT-PCR was performed with commercially available kit (Promega, WI, USA) following the manufacturer's protocol. The quantitative estimation of RT-PCR products was accomplished by densitometric analysis of the bands after agarose gel electrophoresis, and the results were expressed as the ratio between the intensity of *HSPA1A* band to that of the *β-actin* signal in HeLa cells. Total RNA was extracted from yeast cells using the method of Trotter et al [38], and *ACT1* was used as an internal reference for standardizing *SSA3* mRNA expression.

### Western blot analysis

Hsp70 proteins in total cell extracts of HeLa cells were subjected to Western-blot analyses. After heat shock for 1 h at 42°C and recovery at 37°C for 8–16 h, 1×10^6^ HeLa cells were pelleted by centrifugation, washed twice with ice-cold PBS, and lysed on ice in RIPA buffer [30 mM HEPES, 150 mM NaCl, 1% Triton X-100, 0.1% SDS and 1% deoxycholic acid (pH 7.6)], with added proteinase inhibitors, pepstatin A (1.0 µg/ml), aprotinin (3.5 µg/ml), leupeptin (10 µg/ml) and 0.2 mM PMSF. Cell lysates were centrifuged at 3000 g for 15 min at 4°C, and BSA protein assay was performed. Protein samples (100 µg) were heated for 5 min at 100°C and were separated on 10% SDS-PAGE and transferred to PVDF membranes (Millipore, American) in Tris-glycine buffer (pH 8.5) plus 20% methanol. The membranes were blocked overnight in 5% non-fat dry milk in Tris-buffer containing 0.1% Tween-20 and then washed with Tris-buffer. The blots were incubated for 2 h at room temperature with rabbit anti-human Hsp70 polyclonal antibody (Abcam, U.K.) diluted 1∶ 200 in Tris-buffer. The blots were washed, and then incubated with AP-conjugated goat anti-rabbit secondary antibodies (Abcam, U.K.) at 1∶ 500 dilution. The signals were visualized with BCIP/NBT (Millipore, American) system. *β-actin* was used as the internal control.

### Chromatin immunoprecipitation (ChIP)

After appropriate heat-shock treatments, HeLa cells were crosslinked with 2% (v/v) formaldehyde for 10 min at 37°C, and then lysed in SDS lysis buffer [1% SDS, 10 mM EDTA, 50 mM Tris-HCl, pH 8.1] with protease inhibitors. Yeast cells were crosslinked with 1% (v/v) formaldehyde for 10 min at 37°C, and then lysed in 700 µl of lysis buffer [50 mM Hepes/KOH (pH 7.5), 140 mM NaCl, 1 mM EDTA, 1% (v/v) Triton X-100 and 0.1% (v/v) sodium deoxycholate] with protease inhibitors. The sonicated lysates were processed using a ChIP assay kit, essentially as described by the manufacturer (Upstate Biotechnology, Lake Placid, NY). Immunoprecipitation was carried out with antibodies against the N-terminal of hElp3. Input and immunoprecipitated DNA were analyzed by PCR. The primers used can be found in Table 1.

### Statistical analysis

Data are presented as means ± standard deviation (SD) and analyzed with SPSS 11.0. The data among the groups were compared using one-way analysis of variance (ANOVA). P values less than 0.05 were regarded as statistically significant.
